# Combining MLC and SVM Classifiers for Learning Based Decision Making: Analysis and Evaluations

**DOI:** 10.1155/2015/423581

**Published:** 2015-05-21

**Authors:** Yi Zhang, Jinchang Ren, Jianmin Jiang

**Affiliations:** ^1^School of Computer Software, Tianjin University, Tianjin 300072, China; ^2^Centre for Excellence in Signal and Image Processing, University of Strathclyde, Glasgow G1 1XW, UK; ^3^School of Computer Science and Software Engineering, Shenzhen University, Shenzhen 518060, China

## Abstract

Maximum likelihood classifier (MLC) and support vector machines (SVM) are two commonly used approaches in machine learning. MLC is based on Bayesian theory in estimating parameters of a probabilistic model, whilst SVM is an optimization based nonparametric method in this context. Recently, it is found that SVM in some cases is equivalent to MLC in probabilistically modeling the learning process. In this paper, MLC and SVM are combined in learning and classification, which helps to yield probabilistic output for SVM and facilitate soft decision making. In total four groups of data are used for evaluations, covering sonar, vehicle, breast cancer, and DNA sequences. The data samples are characterized in terms of Gaussian/non-Gaussian distributed and balanced/unbalanced samples which are then further used for performance assessment in comparing the SVM and the combined SVM-MLC classifier. Interesting results are reported to indicate how the combined classifier may work under various conditions.

## 1. Introduction

Maximum likelihood classification (MLC) is one of the most commonly used approaches in signal classification and identification, which has been successfully applied in a wide range of engineering applications including classification for digital amplitude-phase modulations [1], remote sensing [2], genes selection for tissue classification [3], nonnative speech recognition [4], chemical analysis in archaeological applications [5], and speaker recognition [6]. On the other hand, support vector machines (SVM) have attracted much increasing attention, which can be found in almost all areas when prediction and classification of signal are required, such as scour prediction on grade-control structure [7], fault diagnosis [8], EEG signal classification [9], and fire detection [10] as well as road sign detection and recognition [11].

Based on the principles of Bayesian statistics, MLC provides a parametric approach in decision making where the model parameters need to be estimated before they are applied for classification. On the contrary, SVM is a nonparametric approach, where the theoretic background is supervised machine learning. Due to the differences of these two classifiers, their performance appears to be much different. Taking the application in remote sensing, for example, in Pal and Mather [12] and Huang et al. [13], it is found that SVM outperforms MLC and several other classifiers. In Waske and Benediktsson [14], SVM produces better results from SAR images, yet in most cases it generates worse results than MLC from TM images. In Szuster et al. [15], SVM only yields slightly better results than MLC for land cover analysis. As a result, detailed assessments as on what conditions SVM outperforms or appears inferior to MLC are worth further investigation.

Furthermore, there becomes a trend to combine the principle of MLC, Bayesian theory, with SVM for improved classification. In Ren [16], Bayesian minimum error classification is applied to the predicted outputs of SVM for error-reduced optimal decision making. Similarly, in Vong et al. [17], Bayesian decision theory is applied in SVM for imbalance measurement and feature optimization for improved performance. In Vega et al. [18], Bayesian statistics are combined with SVM for parameter optimization. In Hsu et al. [19], Bayesian inference is applied to estimate the hyperparameters used in SVM learning to speed up the training process. In Foody [20], relevance support machine (RVM), a Bayesian extension of SVM, is proposed which enables an estimate of the posterior probability of class membership where conventional SVM fail to do so. Consequently, in-depth analysis of the two classifiers is desirable to discover their pros and cons in machine learning.

In this paper, analysis and evaluations of SVM and MLC are emphasized, using data from various applications. Since the selected data satisfy certain conditions in terms of specific sample distributions, we aim to find out how the performance of the classifiers is connected to the particular data distributions. As a consequence, the work and the results shown in the paper are valuable for us to understand how these classifiers work, which can then provide insightful guidance as how to select and combine them in real applications.

The remaining parts of the paper are organized as follows.  Section 2 introduces the principles of the two classifiers.  Section 3 describes data and methods that have been used, where experimental results and evaluations are analyzed and discussed in Section 4. Concluding remarks are given in Section 5.

## 2. MLC and SVM Revisited

In this section, the principles of the two classifiers, SVM and MLC, are discussed. By comparing their theoretic background and implementation details, the two classifiers are characterized in terms of their performances during the training and testing processes. This in turn has motivated our work in the following sections.

### 2.1. The Maximum Likelihood Classifier (MLC)

Let **x**_*i*_ = (*x*_*i*,1_, *x*_*i*,2_,…, *x*_*i*,*N*_)^*T*^,  *i* ∈ [1, *M*], be a group of* N*-dimensional features, derived from *M* observed samples, and *y*_*i*_ ∈ [1, *C*] denotes the class label associated with **x**_*i*_; that is, in total we have *C* classes denoted as *ω*_*c*_, *c* ≥ 2. The basic assumption of MLC is that for each class of data the feature space satisfies specified distributions, usually Gaussian, and also the samples are independent of each other. To this end, the likelihood (probability) for samples within the* k*th class, *ω*_*k*_, is given as follows:

(1) px ∣ ωc=12πN/2Sc1/2exp⁡−12x−μcTSc−1x−μc,

where ***μ***_*c*_ and **S**_*c*_, respectively, denote the mean vector and covariance of all *N*_*c*_ samples within *ω*_*c*_, which can be determined using maximum likelihood estimation as

(2) μcNc−1∑i=1Ncxi,Sc=Nc−1∑i=1Ncxi−μcxi−μcT.

For a given sample **x**_*i*_, the probability it belongs to class *ω*_*c*_ can be denoted as *p*(*ω*_*c*_∣**x**_*i*_). The class *c* that **x**_*i*_ is determined to be within is then decided by

(3) $$\begin{array}{c} f_{MLC}\left(\;x_{i}\;\right)=\underset{c}{\mathrm{argmax}}\left(\;p\left(\;\omega_{c}\;|\;x_{i}\;\right)\;\right). \end{array}$$

Based on Bayesian theory, we have

(4) $$\begin{array}{c} p\left(\;\omega_{c}\;|\;x_{i}\;\right)=\frac{p\left(\omega_{c}\right)p\left(x_{i}\;|\;\omega_{c}\right)}{p\left(x_{i}\right)}. \end{array}$$

Since *p*(**x**_*i*_) is a constant in (4) when **x**_*i*_ is given, (3) can be rewritten as

(5) $$\begin{array}{c} f_{MLC}\left(\;x_{i}\;\right)=\underset{c}{\mathrm{argmax}}\left(\;p\left(\;\omega_{c}\;\right)p\left(\;x_{i}\;|\;\omega_{c}\;\right)\;\right). \end{array}$$

Applying logarithm operation to the right side of (5), also letting *g*_*c*_(**x**) = ln*p*(**x**∣*ω*_*c*_) + ln*p*(*ω*_*c*_) be the discriminating function, (5) becomes

(6) fMLCxi=argmaxcgcxi,

(7) gcx=−12x−ucTSc−1x−uc−N2ln2π−12lnSc+lnpωc.

Again we can ignore the constant in (7) and simplify the discriminating function as

(8) gcx−12x−ucTSc−1x−uc−12lnSc+lnpωc=xTWcx+wcTx+ηc,

where **W**_*c*_ = −(2**S**_*c*_)^−1^, **w**_*c*_ = **S**_*c*_^−1^ **u**_*c*_, and *η*_*c*_ = −2^−1^ **u**_*c*_^T^ **S**_*c*_^−1^ **u**_*c*_ − 2^−1^ln | **S**_*c*_ | + ln*p*(*ω*_*c*_).

As can be seen, *g*_*c*_(**x**) is now a quadratic function of **x** depending on three parameters, that is, **u**_*c*_, **S**_*c*_, and *p*(*ω*_*c*_). When the class *c* is specified, these parameters are determined; hence the quadratic function only depends on the class *c* and the input sample **x**. Also it is worth noting that the third item *η*_*c*_ is actually a constant.

In a particular case when *p*(*ω*_*c*_) is a constant for all *c*, that is, the prior probability that a sample belongs to one of the classes is equal, ln*p*(*ω*_*c*_) in (8) can be ignored; hence the discriminating function is rewritten as

(9) $$\begin{array}{c} g_{c}\left(\;x\;\right)=-{\left(\;x-u_{c}\;\right)}^{T}S_{c}^{-1}\left(\;x-u_{c}\;\right)-ln\left|\;S_{c}\;\right|, \end{array}$$

where the scalar 1/2 is also ignored as it makes no difference when (6) is applied for decision making. However, such simplification cannot be made unless we have clear knowledge about the equal distribution of the samples over the *C* classes.

Based on (9), the decision function can be further simplified if the total number of classes is reduced to two, where the two classes are denoted as −1 and 1 and the sign function is introduced for simplicity:

(10) fMLCxisign⁡gMLCxi=−1,if  gMLCxi<0,1,if  gMLCxi>0,gMLCxx−u−TS−−1x−u−−x−u+TS+−1x−u++lnS+S−.

Moreover, in a special case when **S**_+_ = **S**_−_ = **S**, the quadratic decision function in (10) becomes a linear one as

(11) gMLCx=u−−u+TS−1x−2−1u−TS−1u−−u+TS−1u+.

### 2.2. The Support Vector Machine (SVM)

SVM was originally developed for the classification of two-class problem. In Cortes and Vapnik [21], the principles of SVM are comprehensively discussed. Let the two classes be denoted as 1 and −1, similar to the decision function for MLC in (10); the decision function for linear SVM is given by

(12) yi=fSVMxi=1,if  gSVMxi≥1,−1,if  gSVMxi≤−1,gSVMxi=wTxi+b,

where *y*_*i*_ denotes the labeled value for the input sample **x**_*i*_; **w** and *b* are parameters to be determined in the training process.

Note that the decision function in (12) is actually equivalent to the one in (10) if we adjust the scalar for *b*, yet (12) is more feasible as it has increased the decision margin between the two classes from near zero to 2 | **w**|^−1^. By multiplying *y*_*i*_ to both sides of the discriminating function *g*, this can be further simplified as *y*_*i*_ *g*_SVM_(**x**_*i*_) ≥ 1, that is,

(13) $$\begin{array}{c} y_{i}\left(\;w^{T}x_{i}+b\;\right)\geq 1. \end{array}$$

Hence, the optimal hyperplane to separate the training data with a maximal margin is defined by

(14) $$\begin{array}{c} w_{o}^{T}x+b_{o}=0, \end{array}$$

where **w**_*o*_ and *b*_*o*_ are the determined parameters, and the maximal distance becomes 2 | **w**_*o*_|^−1^.

To determine this optimal hyperplane, we need to maximize 2 | **w**|^−1^, or equivalently to minimize 2^−1^ | **w**|^2^, subject to ∀**x**_*i*_, *y*_*i*_(**w** ^*T*^ **x**_*i*_ + *b*) ≥ 1. Using the Lagrangian multipliers, this optimization problem can be solved by

(15) Ωw,b,λi=12w2−∑i=1LλiyiwTxi+b−1s.t.⁡  λi≥0.

Eventually, the parameters **w**_*o*_ and *b*_*o*_ are decided as

(16) wo∑i=1Lλiyixi,b=yi−woTxi,where  λi≠0.

For any nonzero *λ*_*i*_, the corresponding **x**_*i*_ is denoted as one support vector which naturally satisfies *y*_*i*_(**w** ^*T*^ **x**_*i*_ + *b*) = 1. Therefore, **w**_*o*_ is actually the linear combination of all support vectors. Also we have ∑*λ*_*i*_ *y*_*i*_ = 0.

Eventually if we combine (16) with (12), the discrimination function for any test sample **x** becomes

(17) $$\begin{array}{c} g_{SVM}\left(\;x\;\right)=\overset{L}{\underset{i=1}{\sum }}\lambda_{i}y_{i}x_{i}^{T}x+b \end{array}$$

which solely relies on the inner product of the support vector and the test sample.

For nonlinear problems which are not linearly separable, the discrimination function is extended as

(18) gSVMx∑i=1LλiyiϕxiTϕx+b=∑i=1LλiyiKxi,x+b,

where *ϕ* aims to map the input samples to another space, thus making them linearly separable.

Another important step is to introduce the* kernel trick* to calculate the inner product of mapped samples, that is, 〈*ϕ*(**x**_*i*_), *ϕ*(**x**)〉, which avoids the difficulty in determining the mapping function *ϕ* and also the cost for calculation of the mapped samples and their interproduct. Several typical kernels including linear, polynomial, and radial basis function (RBF) are summarized as follows:

(19) $$\begin{array}{c} K\left(\;x_{i},x_{j}\;\right)=\left\{\begin{array}{cc} x_{i}^{T}x_{j} & \text{linear}\\ {\left(x_{i}^{T}x_{j}+1\right)}^{p},\;p>0 & \text{polynomial}\\ \exp\left(-\frac{{\left\|x_{i}-x_{j}\right\|}^{2}}{2\sigma^{2}}\right) & \text{RBF,} \end{array}\right. \end{array}$$

where optimal values for the associated parameters *p* and *σ* are determined automatically during the training process.

Though SVM is initially developed for two-class problems, it has been extended to deal with multiclass classification based on either combination of decision results from multiple two-class classifications or optimization on multiclass based learning. Some useful further readings can be found in [22–24].

### 2.3. Analysis and Comparisons

MLC and SVM are two useful tools for classification problems, where both of them rely on supervised learning in determining the model and parameters. However, they are different in several ways as summarized below.

Firstly, MLC is a parametric approach which has a basic assumption that the data satisfy Gaussian distribution. On the other contrary, SVM is a nonparametric approach and it has no requirement on the prior distribution of the data, yet various kernels can be empirically selected to deal with different problems.

Secondly, for MLC the model parameters, ***μ***_*c*_ and **S**_*c*_, can be directly estimated using the training data before they are applied for testing and prediction. However, SVM relies on supervised machine learning, in an iterative way, to determine a large amount of parameters including **w**_*o*_, *b*_*o*_, all nonzero *λ*_*i*_, and their corresponding support vectors.

Thirdly, MLC can be straightforward applied to two-class and multiclass problems, yet additional extension is needed for SVM to deal with multiclass problem as it is initially developed for two-class classification.

Finally, a posterior class probabilistic output for the predicted results can be intuitively generated from MLC, which is a valuable indicator for classification to show how likely a sample belongs to a given class. For SVM, however, this is not an easy task though some extensions have been introduced to provide such an output based on the predicted value from SVM. In Platt [25], a posterior class probability *p*_*i*_ is estimated by a sigmoid function as follows:

(20) $$\begin{array}{c} p_{i}=P\left(\;y=1\;|\;x_{i}\;\right)\approx \frac{1}{1+\exp\left(Ag_{SVM}\left(x_{i}\right)+B\right)}. \end{array}$$

The parameters *A* and *B* are determined by solving a regularized maximum likelihood problem as follows:

(21) A∗,B∗=argminA,B−∑i=1Ltilog⁡pi+1−tilog⁡1−pi,ti=1+N12+N1,if  yi=1,12+N−1,if  yi=−1,

where *N*_1_ and *N*_−1_ denote the number of support vectors labeled in classes 1 and −1, respectively.

In addition, in Lin et al. [26] Platt's approach is further improved to avoid any numerical difficulty, that is, overflow or underflow, in determining *p*_*i*_ in case *E*_*i*_ = *Ag*_SVM_(**x**_*i*_) + *B* is either too large or too small:

(22) $$\begin{array}{c} p_{i}=\left\{\begin{array}{cc} {\left(1+e^{-E_{i}}\right)}^{-1}, & \text{if}\;\;E_{i}\geq 0,\\ e^{E_{i}}{\left(1+e^{E_{i}}\right)}^{-1}, & \text{otherwise.} \end{array}\right. \end{array}$$

Although there are significant differences between SVM and MLC, the probabilistic model above has uncovered the connection between these two classifiers. Actually, in Franc et al. [27] MLC and SVM are found to be equivalent to each other in linear cases, and this can also be convinced by similar decision functions in (10) and (12).

## 3. Data and Methods

In this paper, analysis and evaluations of SVM and MLC are emphasized, using data from various applications. Since the selected data satisfy certain conditions in terms of specific sample distributions, we aim to find out how the performance of the classifiers is connected to the particular data distributions. As a consequence, the work and the results shown in the paper are valuable for us to understand how these classifiers work, which can then provide insightful guidance as how to select and combine them in real applications.

### 3.1. The Datasets

In our experiments, four different datasets, SamplesNew, svmguide3, sonar, and splice, are used. Among these four datasets, SamplesNew is a dataset of suspicious microclassification clusters extracted from [16] and svmguide3 is a demo dataset of practical SVM guide [28], whilst sonar and splice datasets come from the UCI repository of machine learning databases [29]. Actually, two principles are applied in selecting these datasets: the first is how balanced the samples are distributed over two classes, and the second is whether the feature distributions are Gaussian-alike. As can be seen, the first two datasets are severely imbalanced, especially the first one, as there are far more data samples in one class than those in another class. On the other hand, the last two datasets are quite balanced. Regarding feature distributions, SamplesNew and svmguide3 are apparently non-Gaussian distributed, yet the other two, sonar and splice, show approximately Gaussian characteristics when the variables are separately observed. This is also validated by the determined Pearson's moment coefficient of skewness below [30], where *μ*_*i*_ and *σ*_*i*_ are the mean and standard deviation for the *i*th dimension of the dataset and *E*(·) refers to mathematical expectation. When the skewness coefficients are determined for each data dimension, the maximum, the minimum, and the average skewness coefficients are obtained and shown in Table 1 for comparisons:

(23) $$\begin{array}{c} S_{i}=\frac{E{\left(x_{i}-\mu_{i}\right)}^{3}}{\sigma_{i}^{3}}. \end{array}$$

### 3.2. The Approach

In our approach, a combined classifier using SVM and MLC is applied, which contains the following three stages. In Stage 1, SVM is used for initial training and classification. For the correctly classified results in SVM, these are employed in Stage 2, where MLC is applied for probability-based modeling. The probability-based models are then utilized in Stage 3 for improved decision making and better classification. Details of these three stages are discussed as follows.

**Stage 1 (SVM for initial training and classification).**

The open source library libSVM [28] is used for initial training and classification of the aforementioned four datasets, and both the linear and the Gaussian radial basis (RBF) kernels are tested. For each group of datasets, all the data are normalized to [−1,1] before SVM is applied. Through 5-fold cross validation, the best group of parameters, including the cost and the gamma value, are optimally determined. Eventually, the optimal parameters are used for classification of our datasets.

In our experiments, the training ratios are set at three different levels, that is, 80%, 65%, and 50%. Basically, there is no overlap between training data and testing data. At a given training ratio, the training data is randomly selected and repeated five times, which leads to 5 groups of test results generated. Finally, the average performance over these five experiments is used for comparisons.

**Stage 2 (using MLC for probability-based modeling).**

For those correctly classified samples, which lie in two classes, that is, class 0 and class 1, they are taken to decide two probability-based models, in a way as discussed in MLC. In other words, for samples correctly classified in class 0, they are used to determine the mean vector and the corresponding covariance matrix within class 0. On the other hand, samples which are correctly classified in class 1 are used to determine the mean vector and the corresponding covariance matrix within class 1. Note that not all samples in class 0 or class 1 are used in calculating the related MLC models, as those which cannot be correctly classified by SVM are treated as outliers and ignored in MLC modeling for robustness.

After MLC modeling, for each sample **x**, the associated likelihoods that it belongs to the two classes are recalculated and denoted as *p*_0_(**x**) and *p*_1_(**x**). As a result, the decision for classification is simplified as

(24) $$\begin{array}{c} f_{MLC}\left(\;x\;\right)=\left\{\begin{array}{cc} 1, & \text{if}\;\;p_{1}\left(x\right)-p_{0}\left(x\right)>\tau ,\\ 0, & \text{otherwise,} \end{array}\right. \end{array}$$

where *τ* is a threshold to be optimally determined to generate the best classified results. Please note that the likelihoods (or probability values) here can also be taken as a probabilistic output of the SVM.

**Stage 3 (improved classification).**

With the estimated MLC models and the optimal threshold *τ*, all samples are then rechecked for improved classification, using (23) and the determined likelihoods *p*_0_(**x**) and *p*_1_(**x**), accordingly. Interesting results on these four datasets are given and analyzed in detail in the next section.

## 4. Results and Evaluations

For the four datasets discussed in Section 3, the experimental results are reported and analyzed in this section. Firstly, we discuss results from a combined classifier of MLC and a linear SVM. Then, results from MLC and RBF based SVM are compared. In addition, how different rebalancing strategies affect the performance of unbalanced datasets is also discussed.

### 4.1. Results from a Linear SVM and the MLC

**Figure 1:**
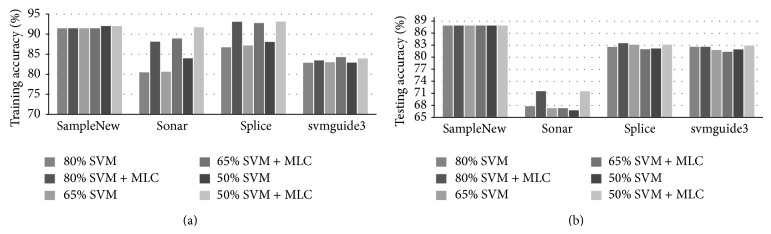
Comparing training (a) and testing results (b) using linear SVM and the combined classifier for the four datasets under three different training ratios.

In this group of experiments, a combined classifier using a linear SVM and the MLC is employed, and the relevant results are presented in Figure 1. In Figure 1, we plot the classification rate as the prediction accuracy with the change of training ratio, that is, the percentage of data used for training. Three training ratios, 80%, 65%, and 50%, are used. Please note that, due to degradation of the covariance matrix, the MLC cannot be used to improve the results for the SampleNew dataset. Consequently, the results from the SVM are taken as the output of the combined classifier. For the other three datasets, the results are summarized and compared as follows.

Firstly, for the three datasets, sonar, splice, and svmguide3, apparently we can see that the combined solution yield significantly improved results in training, especially for the first two datasets. This demonstrates that the combined classifier can indeed achieve more accurate modeling of the datasets. In addition, possibly due to overfitting, the experimental results show that a larger training ratio does not necessarily improve the training performance.

However, the testing results are somehow different. For the sonar dataset, which is balanced and appears nearly Gaussian distributed, the combined classifier yields much improved results in testing, especially when the training ratios are 80% and 50%. Such results are not surprising as the MLC is ideal to model Gaussian-alike distributed datasets. For the splice dataset, which is balanced and also nearly Gaussian distributed, slightly improved testing results are also produced by the combined classifier at training ratios at 80% and 50%, but the testing results at the training ratio of 65% become slightly worse than those from the SVM. For the more challenging svmguide3 dataset, which is unbalanced and non-Gaussian distributed, although the combined classifier yields improved testing results at the training ratio of 50%, the results at the other two training ratios, perhaps due to overfitting, seem inferior to the results from the SVM. Actually, in nature the MLC has difficulty in modeling non-Gaussian distributed datasets, and this explains where the combined classifier makes less contribution to these datasets.

### 4.2. Results from a RBF-Kernelled SVM and the MLC

**Figure 2:**
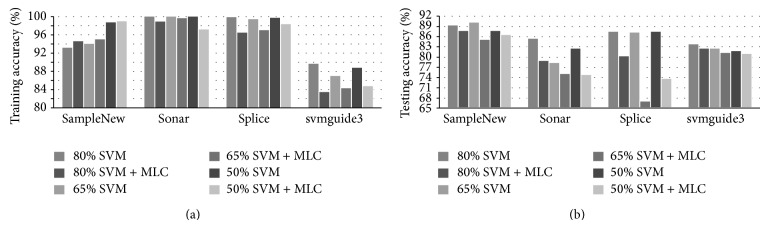
Comparing training (a) and testing results (b) using RBF-kernelled SVM and the combined classifier for the four datasets under three different training ratios.

In this group of experiments, the RBF kernel is used for the SVM in the combined classifier as it is popularly used in various classification problems [16, 22]. For the four datasets we used, again the training results and the testing results under three different training ratios are summarized and given in Figure 2 for comparisons.

First of all, RBF-kernelled SVM (R-SVM) produces much improved results compared to those using linear SVM, especially for the training results. In fact, the combined classifier generates better results than the SVM only in the SampleNew dataset, slightly worse results in sonar and splice datasets, and much degraded results in the svmguide3 dataset.

Regarding testing results, although the combined classifier generates comparable or slightly worse results in the SampleNew dataset and the svmguide3 dataset, R-SVM yields better results in splice dataset and sonar dataset. The reason behind that is that results from the nonlinear kernel in R-SVM cannot be directly refined using MLC. Also, occasionally the results from the combined classifier seem more sensitive to the training ratio, especially for the splice dataset, which is perhaps due to the threshold to be determined which depends more or less on the training data used.

### 4.3. Testing on Rebalanced Data

In this group of experiments, using the challenging dataset svmguide3, how various strategies to rebalance the unbalanced data may affect the classification performance is analyzed. For the unbalanced dataset, samples from one class may be overrepresented compared to those in another class. As a result, we can either oversample the data of minority or subsample the data of majority to balance the number of samples represented in the training set for better modeling of the data. On the other hand, the test samples remain to be unbalanced as it is assumed we have no label information for the test samples.

For oversampling, data samples which are in minority class are randomly duplicated and inserted into the dataset. The replication of data items continues until the entire training set becomes balanced. Different from oversampling, subsampling randomly discards samples from the majority class until the training set achieves balanced. Since the performance may be affected by samples duplicated or discarded, this process is repeated for over 10 times and the average performance is then recorded for comparisons.

**Figure 3:**
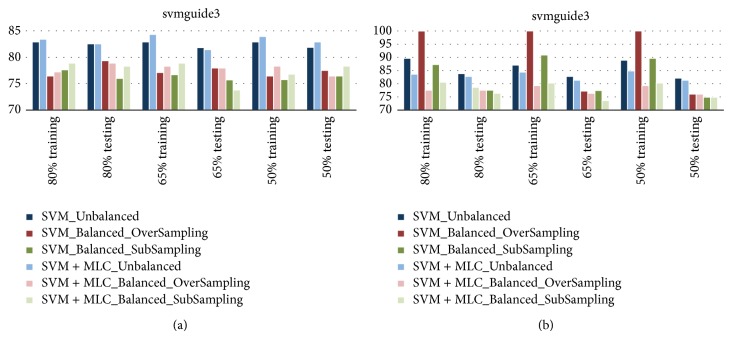
Results of balanced learning for the svmguide3 dataset, using linear SVM (a) and R-SVM (b).

Using three different training ratios at 80%, 65%, and 50%, results of balanced learning for the svmguide3 dataset are summarized in Figure 3. Under a given training ratio, both training results and testing results are presented in groups, where each group contains results from 6 different experimental scenarios. In addition, the results from liner SVM and RBF-kernelled SVM are shown for comparisons as well.

When linear SVM is used, as shown in the first row of Figure 3, surprisingly, the results from unbalanced data are much better than those from balanced data. Also in majority cases, the combined classifier outperforms the SVM classifier in both training and testing, even with balanced learning introduced. The testing results from SVM for balanced learning via oversampling seem better than those from subsampling, yet it seems that the combined classifier produces better results from subsampling based balanced learning.

For RBF-kernelled SVM, apparently, the training results from SVM via oversampling are among the best, though the testing results are inferior to those from unbalanced training. This indicates that the training process has been overfitting in this context. In fact, testing results from the combined classifier are slightly worse than those from the SVM classifier, that is, some degradation. Again, this is caused by the inconsistency of the nonlinear SVM and the linear nature of the MLC.

## 5. Conclusions

SVM and MLC are two typical classifiers commonly used in many engineering applications. Although there is a trend to combine MLC with SVM to provide a probabilistic output for SVM, under what conditions the combined classifier may work effectively needs to be explored. In this paper, comprehensive results are demonstrated to answer the question above, using four different datasets. First of all, it is found that the combined classifier works under certain constraints, such as a linear SVM, balanced dataset, and near Gaussian-distributed data. When a RBF-kernelled SVM is used, the combined classifier may produce degraded results due to the inconsistency between the nonlinear kernel in SVM and linear nature of MLC. In addition, for a challenging dataset, balanced learning may improve the results of training but not necessarily the testing results. The reason behind that is that the combined SVM-MLC classifier works on three assumptions, that is, Gaussian distributed, interclass separable, and model consistency between training data and testing data. Although the third assumption is true in most cases, the precondition of separable Gaussian distributed data is rather a strict constraint for data and is rarely satisfied. As a result, this introduces a fundamental difficulty in combining these two classifiers. However, under certain circumstances, the combined classifier indeed can significantly improve the classification performance. It is worth noting that when more groups are introduced in modelling a given dataset the efficacy can be severely degraded due to the inconsistency of statistical distribution between groups. Future work will focus on combining other classifiers such as neural network for applications in medical imaging [31–33] and recognition and classification tasks [34, 35].

## Figures and Tables

**Table 1:** Four datasets used in our experiments.

Dataset	Features	Balance status	Distribution of feature values	Number of samples (class 0/class 1)	Skewness coefficients		
					Max	Min	Mean
SamplesNew	39	Unbalanced	Non-Gaussian Approx.	748 (115/633)	7.577	−3.063	2.343
svmguide3	21	Unbalanced	Non-Gaussian Approx.	1284 (947/337)	10.074	−4.653	2.181
Sonar	31	Balanced	Approx. Gaussian	209 (97/102)	1.123	−1.019	0.214
Splice	60	Balanced	Approx. Gaussian	1269 (653/616)	0.672	−0.490	−0.016
